# Characterization of the complete plastid genome and phylogenetic analysis of *Oreocharis argyreia* var. *angustifolia* (Gesneriaceae)

**DOI:** 10.1080/23802359.2023.2270207

**Published:** 2023-10-27

**Authors:** Guoliang Xu, Zilin Li, Zhihui Chen, Shiou Yih Lee, Xiaoli Kong

**Affiliations:** a Jiangxi Provincial Management Bureau for, Jiulian Mountain National Natural Reserve, Jiangxi, Longnan, China; b School of Life Science, State Key Laboratory of Biocontrol and Guangdong Provincial Key Laboratory of Plant Resource, Sun Yat-sen University, Guangdong, Guangzhou, China; c INTI International University, Nilai, Negeri Sembilan, Malaysia; d Guangxi Gaofeng State Owned Forest Farm, Guangxi, Nanning, China

**Keywords:** Genetic resources, Jiulian Mountain national nature reserve, lamiales, next-generation sequencing, phylogenomics

## Abstract

*Oreocharis argyreia* var. *angustifolia* of Gesneriaceae is widely distributed in South China, including Guangdong, Guangxi, Hunan, and Jiangxi provinces. However, genetic information of this species is limited, further contributing to the taxonomic complications surrounding this species. Thus, in this study, we assembled and characterized the complete chloroplast genome of *O. argyreia* var. *angustifolia* as a genomic resource for future studies. The complete plastid genome was 154,675 bp in size, with a pair of inverted repeat regions of 25,329 bp each, separating the 85,977-bp large and 18,040-bp small single copy regions. A total of 131 genes were predicted, consisting of 86 protein-coding, 37 tRNA, and eight rRNA genes. The overall GC content was 37.6%. Phylogenetic analysis based on 79 shared unique CDS resulted in a fully resolved phylogenetic tree using both the maximum likelihood and Bayesian inference methods. Based on current circumscription, both methods indicated that *Oreocharis* is monophyletic; *O. argyreia* var. *angustifolia* diverged after *O. chienii*, which then followed by the divergence of the other three species included namely, *O. continifolia*, *O. esquirolii*, and *O. mileensis*. The genomic data obtained will be useful for future studies on the phylogenetics and evolution of Gesneriaceae.

## Introduction

*Oreocharis* of the family Gesneriaceae has been recorded to be made up of approximately 139 species around the world (POWO Plants of the World Online 2022), while most of them are endemic to China (Xu et al. 2017). Similar to orchids, members of Gesneriaceae, including *Oreocharis*, are considered one of the important plant groups for evaluating local ecological environments, and therefore are of significant scientific and economic value (Li et al. 2018). *Oreocharis argyreia* var. *angustifolia* Pan 1987, an endemic herb native to China, was first found in the Guangxi, China (Wang et al. 1990). Lately, it has been discovered to grow along the border of southeast Hunan, southwest Jiangxi provinces, as well as Ruyuan of Guangdong (Fan et al. 2014; Mou et al. 2019; http://gccc.gcib.cn).

**Figure 1.**
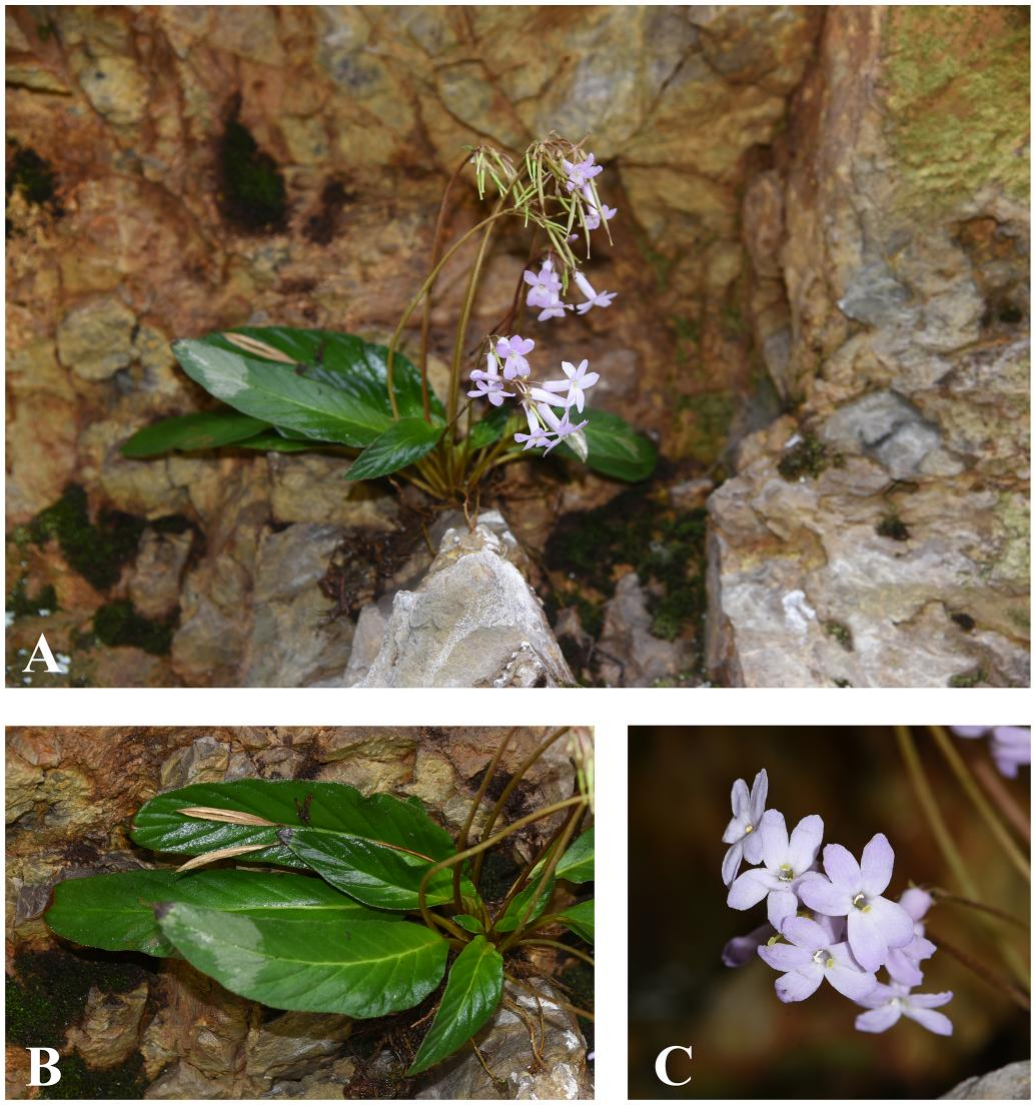
*Oreocharis argyreia* var. *angustifolia* Growing in its natural habitat at Mount Jiulian of Longnan County, Jiangxi Province of China. (A) the whole plant; (B) leaf; (C) corolla. Photos by G. Xu.

*Oreocharis argyreia* var. *angustifolia* comes with an attractive light bluish violet flower (Figure 1). When compared to *O. argyreia* var. *argyreia* Pan 1987, *O. argyreia* var. *angustifolia* has a linear-lanceolate to oblanceolate leaf blade and glabrous ovary while the leaf shape of *O. argyreia* var. *argyreia* is elliptic to ovate and comes with pubescent ovary (Wang et al. 1990). Taxonomic classification in *Oreocharis* remains challenging due to its species richness (Möller et al. 2016). Despite attempts at species delimitation of *Oreocharis* using morphological characteristics aided by geographical and molecular (i.e. DNA sequence data from short fragments) evidence, the relationship within *Oreocharis* and the molecular placement of *O. argyreia* var. *angustifolia* were only partly resolved at a nuclear level based on 573 nuclear orthologous genes (Kong et al. 2022; Yang et al. 2022); studies on the phylogenetic analysis of *Oreocharis* that used plastid gene sequence were in form of a combined dataset of *trn*L-*trn*F and the internal transcribed spacer (ITS) gene sequences, while the relationship in *Oreocharis* is still dubious at plastid level (Wang et al. 2010; Yang et al. 2020). We believe that the lack of informative sites in the DNA fragment dataset has hindered the understanding of this species at the genetic level. Thus, in this study, we sequenced and characterized the complete plastid genome (plastome) of *O. argyreia* var. *angustifolia* and reconstructed a phylogenetic tree to reveal its molecular placement among related species.

## Materials and methods

Fresh leaves of *O. argyreia* var. *angustifolia* were obtained from a natural population in Mount Shixia of Longnan town (24°55’37.79”, 114°51’5.46”). A voucher specimen (collection number: JLSXGL20220512) has been deposited in the Herbarium of Jiulian Mountain National Nature Reserve (contact: Guoliang Xu; email: zxuguoliang@163.com). As the species is not listed as a protected species, no permit is required for specimen collection.

Total genomic DNA was extracted using the cetyltrimethylammonium bromide (CTAB) method (Doyle and Doyle 1990). A 300-bp genomic library was constructed using the TruSeq DNA Sample Prep Kit (Illumina, USA), and sequencing was performed on an Illumina Novaseq platform. Plastome assembly was carried out on the generated 150-bp paired-end raw data using GetOrganelle v1.7.7.0 (Jin et al. 2020) based on default parameters. Gene annotation was performed using GeSeq v2.03 (Tillich et al. 2017) with the default parameters and was manually checked for errors. The annotated plastome and the structure of the genes that are difficult to annotate, including the cis-splicing and trans-splicing genes, were visualized using CPGView (http://www.1kmpg.cn/cpgview).

To infer the molecular placement of *O. argyreia* var. *angustifolia* among its relatives, phylogenetic reconstruction was carried out based on 79 unique CDS shared among the plastomes of 13 taxa of Gesneriaceae. Two species, *Olea paniculata* (Oleaceae; GenBank accession number: MT560031; Dong et al. 2022) and *Plantago depressa* (Plantaginaceae; GenBank accession number: MK144833; Kwon et al. 2019) were included as outgroups. The CDS were aligned using MAFFT 7.470 (Katoh and Standley 2013) before concatenation. Phylogenetic analysis was conducted using the maximum likelihood (ML) and Bayesian inference (BI) methods *via* the RAxML v8 (Stamatakis 2014) and MrBayes v3.2 (Ronquist et al. 2012) pipelines available in the CIPRES Science Gateway (Miller et al. 2010), respectively. The ML tree was constructed using the general-time reversible (GTR) with gamma distribution (+G) (=GTR + G) nucleotide substitution model, coupled with 1,000 bootstrap replicates. Markov chain Monte Carlo with 2,000,000 generations was used for the BI tree, and sampling was taken at every 100 cycles.

## Results

**Figure 2.**
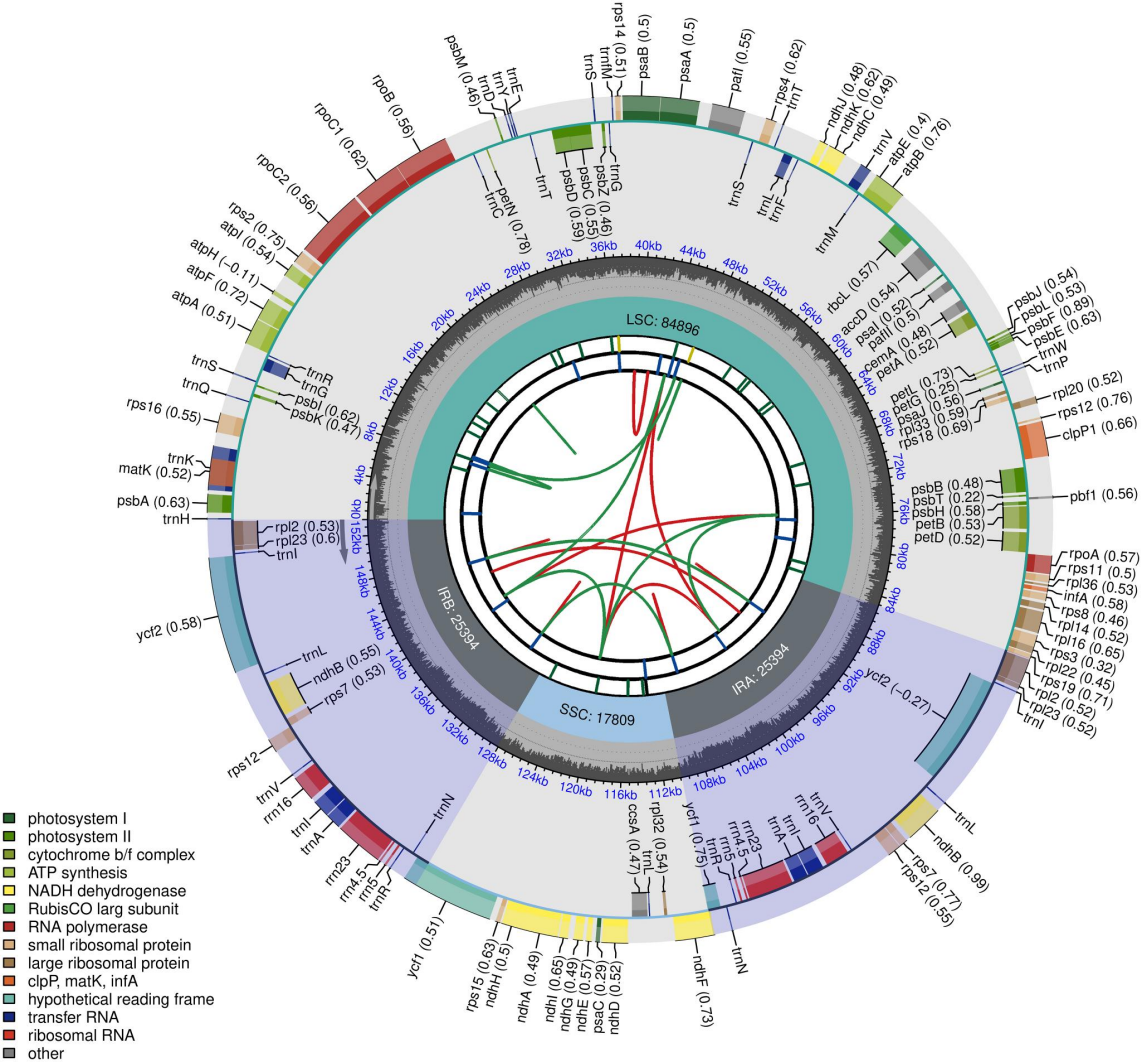
Complete plastome map of *Oreocharis argyreia* var. *angustifolia*. Chloroplast genome map of *Christella dentata.* From the center outward, the first track shows the dispersed repeats, in which the forward (D) and palindromic (P) repeats are connected with red and green arcs. The second track shows the long tandem repeats as short blue bars. The third track shows the short tandem repeats or microsatellite sequences as short bars with different colors that correspond to their repeat unit size: Black: complex repeat; green: repeat unit size = 1; yellow: repeat unit size = 2; purple: repeat unit size = 3; blue: repeat unit size = 4; orange: repeat unit size = 5; red: repeat unit size = 6. The small single-copy, inverted repeat, and large single-copy regions are shown on the fourth track. The GC content along the genome is plotted on the fifth track. The genes are shown on the sixth track, while the optional codon usage bias is displayed in the parenthesis after the gene name. Genes are color-coded by their functional classification (bottom left corner), while the transcription directions for the inner and outer genes are clockwise and anticlockwise, respectively.

With the minimum read mapping depth of 30× and an average read mapping depth of 103.8× (Supplementary Figure 1), the complete plastome of *O. argyreia* var. *angustifolia* (GenBank accession number: ON872365) exhibits a typical quadripartite structure and was 153,493 bp in length (Figure 2). The plastome includes a large single-copy (LSC) region of 84,896 bp, and a small single-copy (SSC) region of 17,809 bp, separated by a pair of 25,394-bp inverted repeat (IR) regions. A total of 131 genes were predicted, including 86 protein-coding, 37 tRNA, and eight rRNA genes. Among them, 13 CDS were cis-splicing genes, of which two contain two introns and 11 contain one intron (Supplementary Figure 2A). The gene structure of the trans-splicing gene, *rps*12 was also identified (Supplementary Figure 2B). The overall GC content was 37.6%.

**Figure 3.**
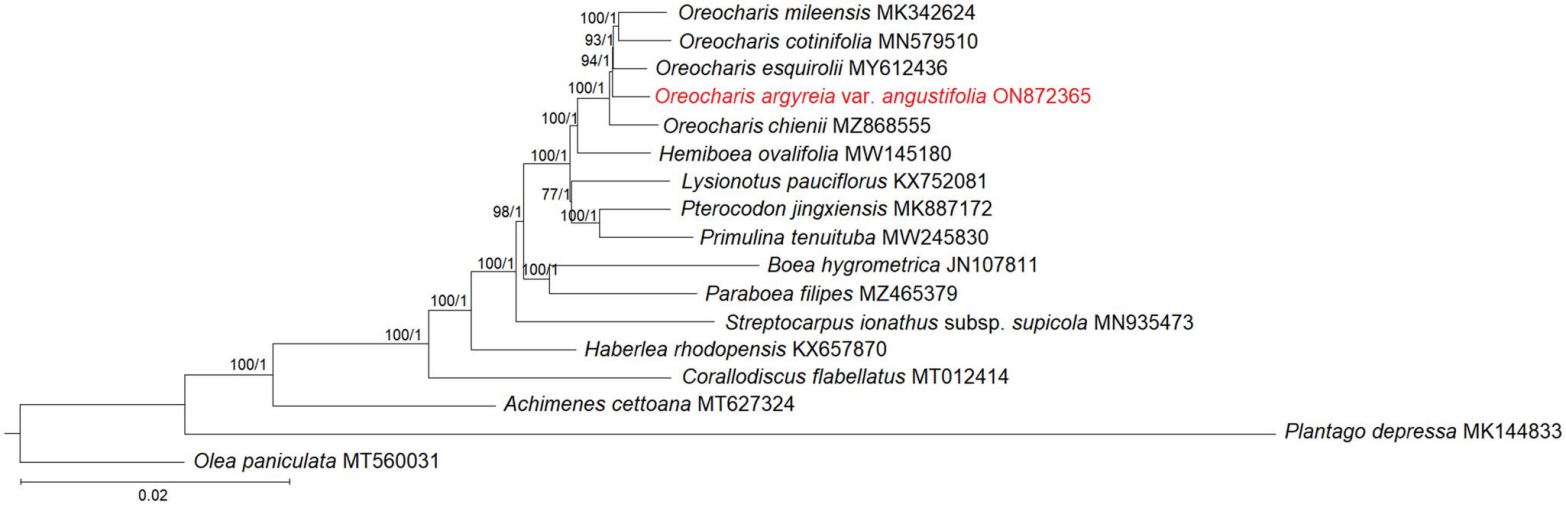
Phylogenetic tree based on the 79 shared unique CDS of 13 selected taxa of Gesneriaceae, with *Olea paniculata* and *Plantago depressa* included as outgroups. Shown next to the nodes are the bootstrap support (BS) and posterior probability (PP) values, in which strong branch support (BS ≥75%, left; PP ≥0.95, right) is indicated with an asterisk (*).

As both the ML and BI trees showed the same topology, only the ML tree was displayed; the posterior probabilities of the BI analysis were incorporated into the ML tree (Figure 3). The phylogenetic relationship among genera in Gesneriaceae was well-resolved (bootstrap support, BS ≥75%; posterior probability, PP ≥0.95). Based on current circumscription, the *Oreocharis* clade is placed sister to *Hemiboea ovalifolia*. Among the four *Oreocharis* species, *O. chienii* was the first to diverge, while followed by *O. argyreia* var. angustifolia and *O. esquirolii*, as well as the other two species, *O. cotinifolia* and *O. mileensis*.

## Discussion

When compared with the available plastomes of three other *Oreocharis* species, all four displayed a similar total number of genes and genome structure (Meng et al. 2019; Gu et al. 2020; Tang et al. 2021). The phylogenetic position of the four species of *Orecharis* included in this study was incongruent when compared to the phylogenetic tree reconstructed using the 573 nuclear orthologous genes (Yang et al. 2022). Based on the nuclear tree, two distinct clades were identified, in which *O. argyreia* var. *angustifolia* is clustered with *O. continifolia* and *O. esquirolii*, while *O. mileensis* was placed in another clade; species of *Oreocharis* is monophyletic when using the complete plastome sequences. Cyto-nuclear discordance is expected in *Oreocharis* as there is evidence of hybridization at the species level (Puglisi et al. 2011). As demonstrated in this study, a resolved tree was reconstructed for Gesneriaceae using the complete plastome sequence; thus, it shows the potential of the plastome tree to provide greater resolution when compared to the short gene sequences, i.e., *trn*L-*trn*F and ITS. Therefore, it is wise to consider the use of the complete plastome sequence to perform phylogenetic reconstruction of *Orecharis* as well as Gesneriaceae, suggesting a considerably enlarged sampling size to uncover the maternal relationship among members of Gesneriaceae.

## Supplementary Material

Supplemental Material

Supplemental Material

## Data Availability

The genome sequence data that support the findings of this study are openly available in GenBank of NCBI at http://www.ncbi.nlm.nih.gov under the accession number ON872365. The associated BioProject, SRA, and BioSample numbers are PRJNA772562, SRR19924869, and SAMN29409930, respectively.
