# Supplementary figures and images for: Characterization of the complete plastid genome and phylogenetic analysis of Oreocharis argyreia var. angustifolia (Gesneriaceae)
Source: Mitochondrial DNA B Resour. 2023 Oct 27;8(10):1137–40. doi: 10.1080/23802359.2023.2270207 (PMC10621260; doi:10.1080/23802359.2023.2270207)

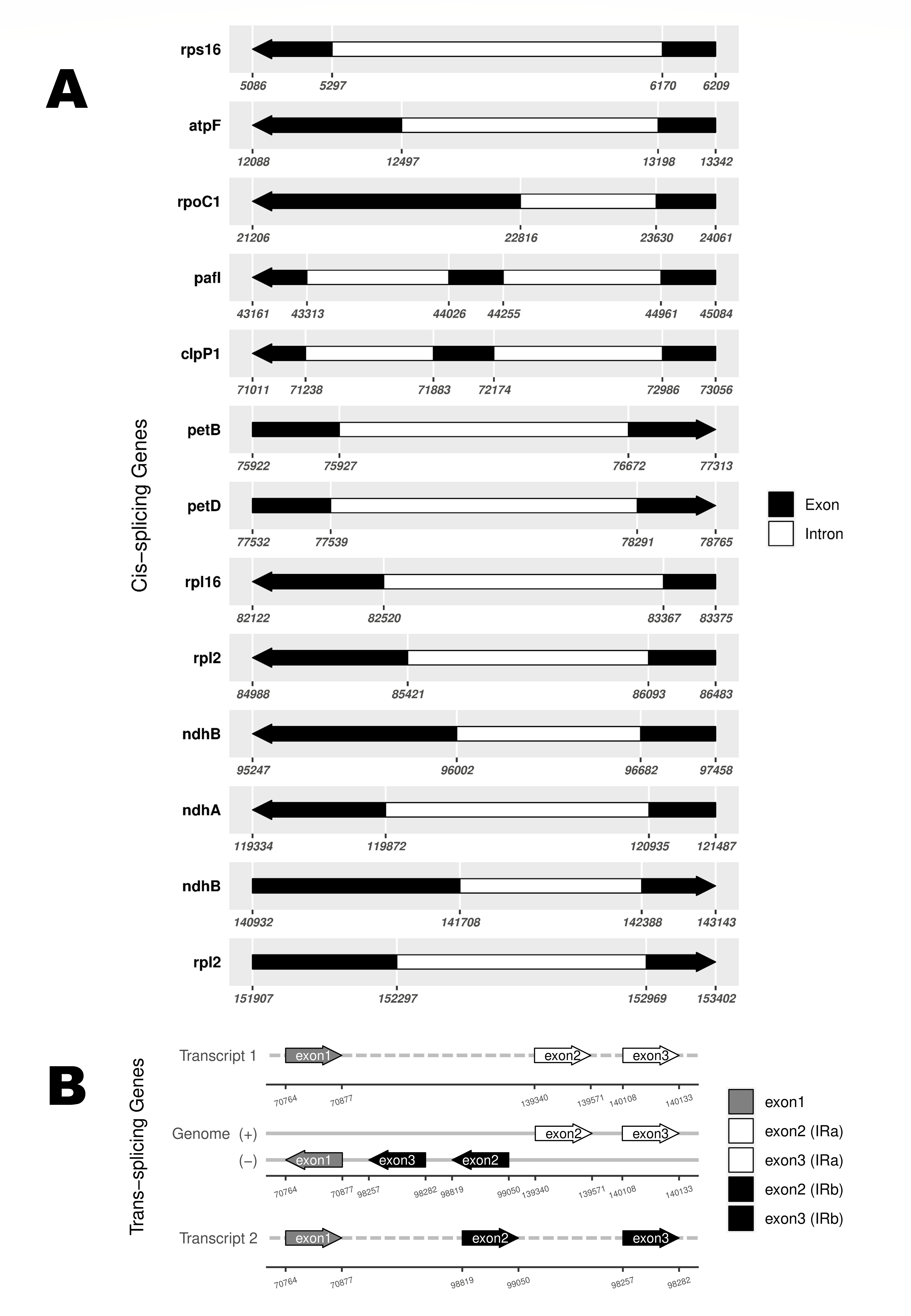

Supplement: Supplemental Material [file TMDN_A_2270207_SM6653.tiff]

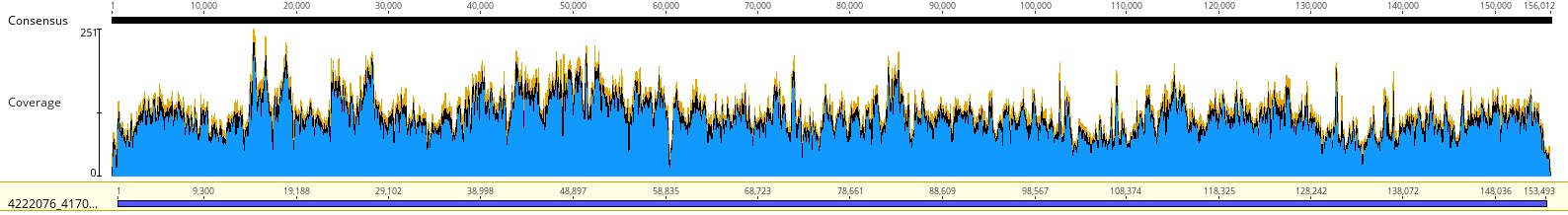

Supplement: Supplemental Material [file TMDN_A_2270207_SM6630.tiff]
